# Cell-ECM interactions control DDR

**DOI:** 10.18632/oncoscience.184

**Published:** 2015-08-10

**Authors:** Ellen Dickreuter, Nils Cordes

**Affiliations:** OncoRay - National Center for Radiation Research in Oncology, Medical Faculty Carl Gustav Carus, Dresden University of Technology, Dresden, Germany

**Keywords:** cell-ecm interactions, DNA repair, integrins

Fundamental to an improvement of cancer cure rates is a better understanding of the underlying molecular mechanisms driving resistance to standard treatments such as radio- and chemotherapy. A powerful and promising approach to sensitize cancer cells to therapy is represented by Paul Ehrlich's old and still up-to-date concept of the “magic bullet”. Reviewing the complexity of the “hallmarks of cancer” provides clarity about the needs to employ pathophysiological models for our examinations and consider multi-targeting concepts [1]. For identifying novel potential cancer targets, we need to unravel the molecular biology of how cancer cells develop, manifest and interact with their microenvironment. These actions are not only promoted by intrinsic genetic and epigenetic alterations but also through a tightly regulated interplay between cancer cells and stromal components documented as key determinants of various hallmarks, including resistance to cell death, sustainability of proliferative signals, and activation of invasion and metastasis.

Among this large plethora of autocrine and paracrine interactions, integrin-mediated adhesion to the extracellular matrix (ECM) is one of the most influencing characteristics eliciting cancer cell therapy resistance. Integrins are transmembrane receptors consisting of one alpha and one beta subunit [2]. Overall, 18 α and 8 β subunits form 24 different integrin cell adhesion receptors. Their dual functionality, ECM binding for structure and for signaling, and their location at subcellular sites pinpoint them as highly interesting molecules. Structurally, a linkage of ECM/integrin/actin cytoskeleton/nuclear membrane/chromatin connects the outside with the inside of the cell [3]. Signaling-wise, outside-in and inside-out communication by integrins significantly contributes to the regulation of cell survival, proliferation, migration or apoptosis.

However what turns integrins into potent cancer targets? First, integrins are overexpressed in different tumor entities relative to the corresponding healthy tissue. Secondly, integrins are critically involved in the prosurvival, promigratory and therapy resistance mechanisms, and third, integrins are surface receptors that are easily druggable and utilizable for imaging. Different preclinical studies convincingly show that inhibition of integrin receptors sensitizes tumor cells to chemo- and radiotherapy [4,5]. As is often the case, clinical trials were unable to recapitulate these findings due to insufficient integrin inhibition. Thus, a new strategy proposed by us and others is to inhibit all β1 integrin associated integrin receptors. As β1 integrins form by far the biggest group of integrin receptors, β1 integrin targeting enables the deactivation of a wide variety of integrins at once. In our case, we used the monoclonal inhibitory anti-β1 integrin antibody AIIB2 in head and neck squamous cell carcinomas (HNSCC). We previously showed that β1 integrins signal via the focal adhesions kinase (FAK)/Cortactin/c-Jun N-terminal kinase 1 (JNK1) signaling axis for increased survival after radiation [4].

Hence, we asked whether there is a connection between β1 integrin signaling and DNA repair. Despite one report on suppression of Bleomycin-induced DNA damage by integrin signaling [6], there was a lack of knowledge about integrin signaling and repair of radiogenic DNA double strand breaks (DSBs). Cells exhibit two pathways for the repair of DSBs: non-homologous end-joining (NHEJ) and homologous recombination. NHEJ is distinguished by classical NHEJ (C-NHEJ), which depends on DNA-PKcs, and backup NHEJ (B-NHEJ), which depends on PARP-1 [7]. In our study using 3D matrix-based cell culture and tumor xenograft models, we focused on the impact of β1 integrins on C- and B-NHEJ [8]. We found increased DSBs after β1 integrin inhibition and radiation in foci and comet assay analysis. Mechanistically, β1 integrins signal via the FAK/JNK1 axis to the repair of radiogenic DSBs. Interestingly, β1 integrin inhibition impaired C-NHEJ whereas B-NHEJ was not influenced. We found enhanced γH2AX- and pDNA-PKcs Thr2609-positive DSBs and decreased Ku70, Rad50 and Nbs1 expression but no PARP-1 modifications after AIIB2-mediated β1 integrin inhibition. Further experiments clearly indicated FAK and JNK1 to be part of a signaling cascade connecting β1 integrins to the C-NHEJ machinery. In terms of the concept of synthetic lethality, we exploited the independence of PARP from β1 integrins by inhibiting PARP with Olaparib. Intriguingly, this combination therapy with AIIB2 and Olaparib resulted in an additive decrease of clonogenic radiation survival, thereby suggesting a new approach to overcome radioresistance in HNSCC.

**Figure 1:**
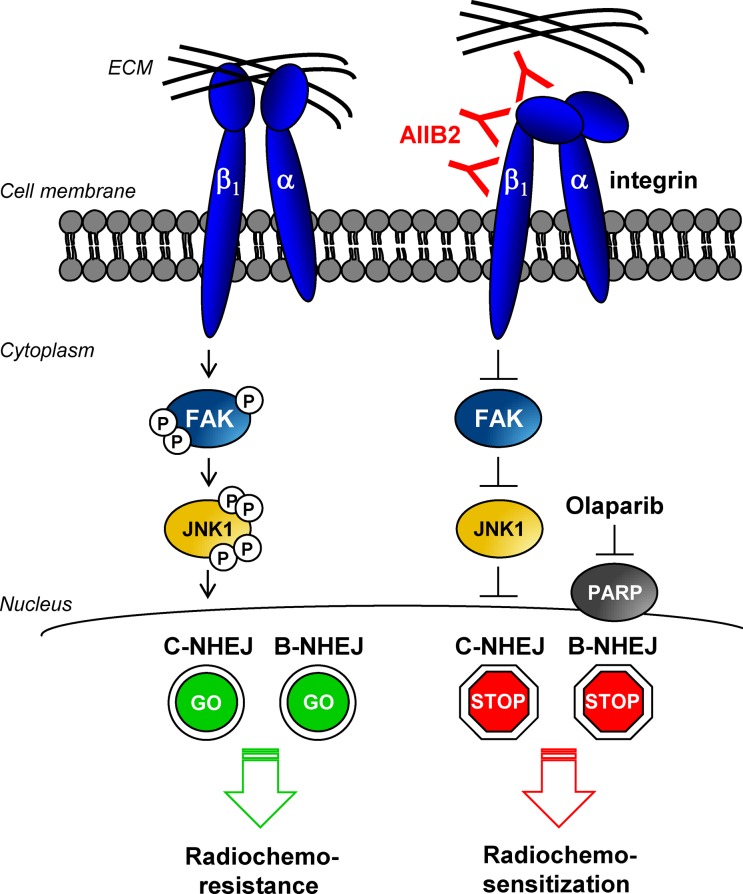
β1 integrin-mediated signaling modulates DNA repair The β1 integrin/ECM interaction leads to activation of FAK and JNK1 supporting DSB repair via DNA-PKcs-dependent C-NHEJ whereas PARP-dependent B-NHEJ remains unaffected. After inhibition of β1 integrins, FAK and JNK1 are inactivated and C-NHEJ activity is reduced. The simultaneous targeting of C- and B-NHEJ via AIIB2 and Olaparib, respectively, leads to additive radiochemosensitization of HNSCC cells suggesting a new strategy to overcome tumor cell therapy resistance.

To our knowledge, this is the first study showing a mechanistic connection between β1 integrin targeting and impaired DNA repair, thus connecting the ECM/integrin interplay with radiation-induced DNA repair processes.

In conclusion, we show that the β1 integrin-mediated adhesion to the ECM co-determines both the repair of radiogenic DSBs and the clonogenic radiation survival. Further characterization of β1 integrin-mediated prosurvival signaling including the interrelation between β1 integrin and homologous recombination is necessary for a safe, effective and reasonable translation into the clinic.
